# Supplementary material for: Heparin-based hydrogel scaffolding alters the transcriptomic profile and increases the chemoresistance of MDA-MB-231 triple-negative breast cancer cells
Source: Biomater Sci. 2020 Feb 13;8(10):2786–96. doi: 10.1039/c9bm01481k (PMC7497406; doi:10.1039/c9bm01481k)
Supplement: Supplementary file 2 [file BM-008-C9BM01481K-s002.zip › Supplementary File 4/HP-B hydrogel + EGF (s)vControl/Pathways/my_analysis.Gsea.1545201170987/HALLMARK_FATTY_ACID_METABOLISM.html]

Details for gene set HALLMARK\_FATTY\_ACID\_METABOLISM[GSEA]

|  || Dataset | expr.class.cls#HYDEGF\_versus\_CONTROL.class.cls#HYDEGF\_versus\_CONTROL\_repos |
| Phenotype | class.cls#HYDEGF\_versus\_CONTROL\_repos |
| Upregulated in class | CONTROL |
| GeneSet | HALLMARK\_FATTY\_ACID\_METABOLISM |
| Enrichment Score (ES) | -0.43442437 |
| Normalized Enrichment Score (NES) | -1.9920224 |
| Nominal p-value | 0.0 |
| FDR q-value | 3.3161763E-4 |
| FWER p-Value | 0.002 |
Table: GSEA Results Summary

  

Fig 1: Enrichment plot: HALLMARK\_FATTY\_ACID\_METABOLISM      
 Profile of the Running ES Score & Positions of GeneSet Members on the Rank Ordered List

  

| PROBE | DESCRIPTION (from dataset) | GENE SYMBOL | GENE\_TITLE | RANK IN GENE LIST | RANK METRIC SCORE | RUNNING ES | CORE ENRICHMENT || 1 | CYP1A1 | na |  |  | 102 | 2.902 | 0.0192 | No |
| 2 | NCAPH2 | na |  |  | 327 | 2.346 | 0.0273 | No |
| 3 | SLC22A5 | na |  |  | 456 | 2.172 | 0.0390 | No |
| 4 | AADAT | na |  |  | 463 | 2.164 | 0.0570 | No |
| 5 | D2HGDH | na |  |  | 703 | 1.905 | 0.0606 | No |
| 6 | GCDH | na |  |  | 940 | 1.721 | 0.0628 | No |
| 7 | ACAA1 | na |  |  | 1222 | 1.561 | 0.0612 | No |
| 8 | ENO3 | na |  |  | 1899 | 1.295 | 0.0367 | No |
| 9 | ECI1 | na |  |  | 2082 | 1.242 | 0.0377 | No |
| 10 | CPT1A | na |  |  | 2216 | 1.207 | 0.0409 | No |
| 11 | MLYCD | na |  |  | 2419 | 1.147 | 0.0400 | No |
| 12 | FASN | na |  |  | 3578 | 0.888 | -0.0132 | No |
| 13 | AOC3 | na |  |  | 3854 | 0.837 | -0.0205 | No |
| 14 | ODC1 | na |  |  | 3955 | 0.820 | -0.0188 | No |
| 15 | IL4I1 | na |  |  | 4181 | 0.785 | -0.0240 | No |
| 16 | LTC4S | na |  |  | 4210 | 0.780 | -0.0188 | No |
| 17 | ACADVL | na |  |  | 4708 | 0.706 | -0.0389 | No |
| 18 | DHCR24 | na |  |  | 4964 | 0.657 | -0.0467 | No |
| 19 | CPT2 | na |  |  | 5037 | 0.647 | -0.0451 | No |
| 20 | CEL | na |  |  | 5266 | 0.611 | -0.0518 | No |
| 21 | AUH | na |  |  | 5491 | 0.577 | -0.0587 | No |
| 22 | ACOT2 | na |  |  | 5566 | 0.568 | -0.0578 | No |
| 23 | PTPRG | na |  |  | 6768 | 0.399 | -0.1174 | No |
| 24 | BCKDHB | na |  |  | 6997 | 0.371 | -0.1262 | No |
| 25 | ME1 | na |  |  | 7136 | 0.354 | -0.1304 | No |
| 26 | ACADS | na |  |  | 7614 | 0.288 | -0.1530 | No |
| 27 | UROS | na |  |  | 7943 | 0.250 | -0.1681 | No |
| 28 | MGLL | na |  |  | 8440 | 0.196 | -0.1924 | No |
| 29 | ACSS1 | na |  |  | 8530 | 0.182 | -0.1956 | No |
| 30 | HSD17B10 | na |  |  | 9087 | 0.117 | -0.2237 | No |
| 31 | CBR1 | na |  |  | 9495 | 0.076 | -0.2444 | No |
| 32 | IDH3G | na |  |  | 9687 | 0.051 | -0.2540 | No |
| 33 | SDHA | na |  |  | 9749 | 0.043 | -0.2569 | No |
| 34 | GRHPR | na |  |  | 9778 | 0.039 | -0.2580 | No |
| 35 | PCBD1 | na |  |  | 10145 | -0.002 | -0.2772 | No |
| 36 | ACOT8 | na |  |  | 10148 | -0.002 | -0.2773 | No |
| 37 | GPD1 | na |  |  | 10485 | -0.041 | -0.2945 | No |
| 38 | CRAT | na |  |  | 10668 | -0.062 | -0.3036 | No |
| 39 | HIBCH | na |  |  | 11001 | -0.107 | -0.3201 | No |
| 40 | ACSL1 | na |  |  | 11022 | -0.109 | -0.3202 | No |
| 41 | METAP1 | na |  |  | 11040 | -0.112 | -0.3201 | No |
| 42 | CPOX | na |  |  | 11194 | -0.122 | -0.3271 | No |
| 43 | BMPR1B | na |  |  | 11316 | -0.139 | -0.3323 | No |
| 44 | ACADL | na |  |  | 11372 | -0.143 | -0.3340 | No |
| 45 | YWHAH | na |  |  | 11375 | -0.143 | -0.3329 | No |
| 46 | SUCLG2 | na |  |  | 11434 | -0.151 | -0.3346 | No |
| 47 | ACAT2 | na |  |  | 11541 | -0.166 | -0.3388 | No |
| 48 | HADH | na |  |  | 11730 | -0.192 | -0.3470 | No |
| 49 | HMGCS1 | na |  |  | 11862 | -0.213 | -0.3521 | No |
| 50 | AQP7 | na |  |  | 11942 | -0.214 | -0.3544 | No |
| 51 | GPD2 | na |  |  | 12156 | -0.238 | -0.3636 | No |
| 52 | ADSL | na |  |  | 12322 | -0.265 | -0.3700 | No |
| 53 | NBN | na |  |  | 12404 | -0.272 | -0.3719 | No |
| 54 | H2AFZ | na |  |  | 12572 | -0.292 | -0.3782 | No |
| 55 | RETSAT | na |  |  | 12706 | -0.315 | -0.3825 | No |
| 56 | DLST | na |  |  | 12764 | -0.326 | -0.3828 | No |
| 57 | PTS | na |  |  | 12846 | -0.335 | -0.3842 | No |
| 58 | ACO2 | na |  |  | 13111 | -0.355 | -0.3950 | No |
| 59 | HSD17B7 | na |  |  | 13211 | -0.372 | -0.3971 | No |
| 60 | IDH3B | na |  |  | 13523 | -0.417 | -0.4098 | No |
| 61 | ALDH3A2 | na |  |  | 13652 | -0.434 | -0.4129 | No |
| 62 | SDHD | na |  |  | 13743 | -0.448 | -0.4138 | No |
| 63 | NTHL1 | na |  |  | 13970 | -0.483 | -0.4216 | No |
| 64 | ECH1 | na |  |  | 14216 | -0.507 | -0.4301 | Yes |
| 65 | BPHL | na |  |  | 14231 | -0.510 | -0.4266 | Yes |
| 66 | G0S2 | na |  |  | 14238 | -0.511 | -0.4225 | Yes |
| 67 | HSPH1 | na |  |  | 14250 | -0.513 | -0.4188 | Yes |
| 68 | CBR3 | na |  |  | 14258 | -0.514 | -0.4148 | Yes |
| 69 | SUCLA2 | na |  |  | 14341 | -0.527 | -0.4146 | Yes |
| 70 | ECHS1 | na |  |  | 14355 | -0.530 | -0.4108 | Yes |
| 71 | MDH1 | na |  |  | 14491 | -0.555 | -0.4132 | Yes |
| 72 | ENO2 | na |  |  | 14703 | -0.591 | -0.4193 | Yes |
| 73 | ACADM | na |  |  | 14777 | -0.601 | -0.4180 | Yes |
| 74 | PPARA | na |  |  | 14852 | -0.612 | -0.4167 | Yes |
| 75 | VNN1 | na |  |  | 14949 | -0.628 | -0.4165 | Yes |
| 76 | ALDH3A1 | na |  |  | 14974 | -0.629 | -0.4124 | Yes |
| 77 | ALAD | na |  |  | 15047 | -0.642 | -0.4107 | Yes |
| 78 | RAP1GDS1 | na |  |  | 15067 | -0.646 | -0.4063 | Yes |
| 79 | EPHX1 | na |  |  | 15301 | -0.687 | -0.4127 | Yes |
| 80 | ACSL4 | na |  |  | 15387 | -0.701 | -0.4112 | Yes |
| 81 | ADIPOR2 | na |  |  | 15521 | -0.725 | -0.4120 | Yes |
| 82 | ACOX1 | na |  |  | 15588 | -0.740 | -0.4092 | Yes |
| 83 | MDH2 | na |  |  | 15652 | -0.754 | -0.4062 | Yes |
| 84 | ERP29 | na |  |  | 15784 | -0.781 | -0.4064 | Yes |
| 85 | TP53INP2 | na |  |  | 15787 | -0.781 | -0.3999 | Yes |
| 86 | REEP6 | na |  |  | 15838 | -0.790 | -0.3958 | Yes |
| 87 | PDHA1 | na |  |  | 15909 | -0.803 | -0.3927 | Yes |
| 88 | ECI2 | na |  |  | 15984 | -0.815 | -0.3897 | Yes |
| 89 | GSTZ1 | na |  |  | 15993 | -0.817 | -0.3832 | Yes |
| 90 | NSDHL | na |  |  | 16044 | -0.831 | -0.3788 | Yes |
| 91 | SMS | na |  |  | 16045 | -0.831 | -0.3718 | Yes |
| 92 | FH | na |  |  | 16089 | -0.841 | -0.3669 | Yes |
| 93 | DLD | na |  |  | 16317 | -0.903 | -0.3712 | Yes |
| 94 | ALDH9A1 | na |  |  | 16400 | -0.923 | -0.3677 | Yes |
| 95 | ALDOA | na |  |  | 16587 | -0.967 | -0.3692 | Yes |
| 96 | DECR1 | na |  |  | 16731 | -1.006 | -0.3682 | Yes |
| 97 | HSDL2 | na |  |  | 16778 | -1.019 | -0.3620 | Yes |
| 98 | HADHB | na |  |  | 16850 | -1.042 | -0.3569 | Yes |
| 99 | ETFDH | na |  |  | 16947 | -1.071 | -0.3529 | Yes |
| 100 | HCCS | na |  |  | 17006 | -1.091 | -0.3467 | Yes |
| 101 | MCEE | na |  |  | 17148 | -1.127 | -0.3446 | Yes |
| 102 | ACSL5 | na |  |  | 17434 | -1.228 | -0.3491 | Yes |
| 103 | ELOVL5 | na |  |  | 17447 | -1.235 | -0.3393 | Yes |
| 104 | HMGCL | na |  |  | 17468 | -1.244 | -0.3298 | Yes |
| 105 | UBE2L6 | na |  |  | 17509 | -1.262 | -0.3212 | Yes |
| 106 | MAOA | na |  |  | 17610 | -1.299 | -0.3155 | Yes |
| 107 | UROD | na |  |  | 17690 | -1.327 | -0.3084 | Yes |
| 108 | GLUL | na |  |  | 17795 | -1.381 | -0.3022 | Yes |
| 109 | LGALS1 | na |  |  | 17822 | -1.398 | -0.2917 | Yes |
| 110 | EHHADH | na |  |  | 17931 | -1.456 | -0.2850 | Yes |
| 111 | PRDX6 | na |  |  | 18067 | -1.524 | -0.2792 | Yes |
| 112 | BLVRA | na |  |  | 18074 | -1.527 | -0.2666 | Yes |
| 113 | ACAA2 | na |  |  | 18083 | -1.534 | -0.2541 | Yes |
| 114 | APEX1 | na |  |  | 18097 | -1.547 | -0.2417 | Yes |
| 115 | IDI1 | na |  |  | 18098 | -1.547 | -0.2286 | Yes |
| 116 | CRYZ | na |  |  | 18132 | -1.566 | -0.2170 | Yes |
| 117 | SDHC | na |  |  | 18153 | -1.583 | -0.2047 | Yes |
| 118 | ACSM3 | na |  |  | 18178 | -1.597 | -0.1924 | Yes |
| 119 | SUCLG1 | na |  |  | 18207 | -1.604 | -0.1803 | Yes |
| 120 | CCDC58 | na |  |  | 18269 | -1.653 | -0.1695 | Yes |
| 121 | HSD17B11 | na |  |  | 18643 | -1.977 | -0.1724 | Yes |
| 122 | S100A10 | na |  |  | 18646 | -1.987 | -0.1557 | Yes |
| 123 | SERINC1 | na |  |  | 18679 | -2.025 | -0.1402 | Yes |
| 124 | IDH1 | na |  |  | 18690 | -2.035 | -0.1235 | Yes |
| 125 | RDH11 | na |  |  | 18715 | -2.072 | -0.1072 | Yes |
| 126 | PDHB | na |  |  | 18748 | -2.110 | -0.0910 | Yes |
| 127 | UGDH | na |  |  | 18907 | -2.368 | -0.0793 | Yes |
| 128 | HSD17B4 | na |  |  | 19012 | -2.605 | -0.0627 | Yes |
| 129 | GABARAPL1 | na |  |  | 19071 | -2.800 | -0.0420 | Yes |
| 130 | PSME1 | na |  |  | 19078 | -2.824 | -0.0185 | Yes |
| 131 | OSTC | na |  |  | 19104 | -2.920 | 0.0049 | Yes |
Table: GSEA details [plain text format]

  

Fig 2: HALLMARK\_FATTY\_ACID\_METABOLISM      
 Blue-Pink O' Gram in the Space of the Analyzed GeneSet

  

Fig 3: HALLMARK\_FATTY\_ACID\_METABOLISM: Random ES distribution      
 Gene set null distribution of ES for **HALLMARK\_FATTY\_ACID\_METABOLISM**
